# A mixed-binomial model for Likert-type personality measures

**DOI:** 10.3389/fpsyg.2014.00371

**Published:** 2014-05-09

**Authors:** Jüri Allik

**Affiliations:** ^1^Department of Psychology, University of TartuTartu, Estonia; ^2^Estonian Academy of SciencesTallinn, Estonia

**Keywords:** personality measurement models, mixed-binomial model, Likert-scale, NEO Personality Inventory, self- and observer-ratings, response bias, measurement invariance

## Abstract

Personality measurement is based on the idea that values on an unobservable latent variable determine the distribution of answers on a manifest response scale. Typically, it is assumed in the Item Response Theory (IRT) that latent variables are related to the observed responses through continuous normal or logistic functions, determining the probability with which one of the ordered response alternatives on a Likert-scale item is chosen. Based on an analysis of 1731 self- and other-rated responses on the 240 NEO PI-3 questionnaire items, it was proposed that a viable alternative is a finite number of latent events which are related to manifest responses through a binomial function which has only one parameter—the probability with which a given statement is approved. For the majority of items, the best fit was obtained with a mixed-binomial distribution, which assumes two different subpopulations who endorse items with two different probabilities. It was shown that the fit of the binomial IRT model can be improved by assuming that about 10% of random noise is contained in the answers and by taking into account response biases toward one of the response categories. It was concluded that the binomial response model for the measurement of personality traits may be a workable alternative to the more habitual normal and logistic IRT models.

## Introduction

Nearly all currently popular personality questionnaires use multiple items paired with a Likert-type response format in which respondents specify their level of agreement or disagreement with a large number of statements describing multiple personality traits. For example, agreement or disagreement can be expressed on a 5-point scale with the following response categories: 1 = strongly disagree, 2 = disagree; 3 = neutral; 4 = agree, and 5 = strongly agree (Costa and McCrae, 1992; Goldberg et al., 2006). Rensis Likert, who introduced this type of response format (Likert, 1932), was obviously interested in capturing a larger range of agreement rather than that of a binary black and white picture without intermediate shades of gray. Since all items belonging to one scale are assumed to be parallel mini-instruments which replicate each other, it is common practice to sum item raw scores together over a whole range of similar items measuring, for instance, angry hostility or openness to new ideas. In so doing, Likert scaling obviously assumes that distances between each neighbouring response category are equal. The subjective distance from “strongly disagree” to “disagree” is expected to be exactly the same as the distance from “neutral” to “agree.” Otherwise it does not make much sense to compute the mean score for angry hostility, openness to new ideas, or any other personality traits by aggregating scores across items belonging to these subscales. Even if researchers can be more or less confident that their respondents understand the order between response categories, that is that “neutral” is supposed to be stronger than “disagree,” there is no guarantee that the perceived interval between the response categories actually corresponds to the relation between the numerals which are used to label response categories. There is no simple and intuitively transparent test which could show whether subjective intervals between neighbouring response categories are reasonably similar or they are different to such an extent that aggregating them together could lead to substantial errors. This may be one of the main reasons why test constructors very seldom analyse distributions of responses of individual items. Usually it is considered enough to observe the standard deviation and skew of a response, in addition to correlations between items, to decide whether this item can or cannot be included in a personality questionnaire.

Most personality researchers seem to be happy with the idea that the observed response scores on a personality item are determined by some unobserved latent variable (Bollen, 2002; Borsboom, 2005). If a person, for example, endorses a statement like “I have sympathy for others less fortunate than me” then she or he is supposed to have an enduring tendency to be moved, more than average, by others' needs and be inclined to emphasize with the human side of social policies. Thus, if there is an item which is supposed to measure a trait called A6: Tender-Mindedness, then a disposition has to really exist that makes people feel sympathy and concern for others (Costa and McCrae, 1992). The basic idea of the Item Response Theory (IRT) is that a mathematical function exists that describes relationship between the latent variable, representing the trait, and responses on a given item (Lord, 1980; van der Linden and Hambleton, 1997; Reise et al., 2005). This mathematical function is usually called Item Response Curve (IRC; also known as Item Characteristic Curve) or, when the number of response alternatives is not dichotomous, Category Response Curve (CRC) (Reise and Waller, 2009).

The choice of the mathematical function relating latent variables to manifest responses has largely been a matter of convenience, rather than a deliberate theoretical justification. Usually it is just postulated that there is a psychological continuum of latent states which is related to response categories through a process described sufficiently well by a normal distribution (cf., Thurstone, 1927). The normal distribution was chosen to represent IRC or CRC mainly due to its own “good properties” (completely specified by the mean and standard deviation, sum of two normal distributions is again normal distribution etc.), not due to any serious factual evidences. As a proof, normal ogive was often replaced with a more popular logistic function which was computationally less demanding than polynomial approximations required for the normal distribution. However, with a trivial rescaling using a single constant it is possible to make the two-parameter logistic function closely approximate the cumulative normal ogive (Camilli, 1994).

Although there are numerous applications of IRT methods to personality data (Chernyshenko et al., 2001; Maij-de Meij et al., 2008; Samuel et al., 2010; Spence et al., 2012; Stepp et al., 2012) there is still an on-going debate whether IRT can be used as extensively in personality measurement as it is in cognitive measurement (Reise and Widaman, 1999; Chernyshenko et al., 2001; Reise and Henson, 2003). Researchers very seldom report IRT model fit to their personality data at the level of single items since a usual Pearson goodness-of-fit statistic (χ^2^) is often too restrictive to provide a tolerable agreement between empirical data points and model's prediction in the strict statistical sense (Stone and Zhang, 2003; Swaminathan et al., 2007; LaHuis et al., 2011). In order to relieve fitting requirements several nonparametric IRT methods were proposed in which only invariant item ordering is taken into account (Mokken and Lewis, 1982; Scheiblechner, 1999, 2007; Sijtsma and Molenaar, 2002; Meijer and Baneke, 2004). In many cases nonparametric IRT models seem to be clearly superior of normal or logistic response functions (Chernyshenko et al., 2001; Sijtsma and Molenaar, 2002; Reise and Waller, 2003).

It is somewhat surprising that latent variables in IRT models are almost always conceptualized as an infinite set of events or psychological states. Even if to assume that two popular functions, normal and logistic, do not need to extend from minus to plus infinity it is still presumed that a latent variable is isomorphic to a considerable segment cut out from a continuum of real numbers. However, continuous response functions are not the only option. At least logically nothing prevents us to consider latent variables represented by a finite set of events or discrete psychological states. Each of these events or states can occur with a certain probability and combination of these latent events are supposed to determine the choice of an explicit response category, for instance choice of one of the response options on a Likert scale. Of course, there are many discrete probability functions but the binomial distribution comes first to the mind. Indeed, any introductory textbook of probability theory starts with an example of coin-tossing which outcomes can be described by a binomial function. As a matter of fact, it was shown that the Rasch model, one of the basic tools of IRT, fits coin-tossing data very well (Wood, 1978), but it appears that very few have thought about coin-tossing itself, or any other similar process consisting of discrete events, as a prototype for a latent variable.

**Figure 1:**
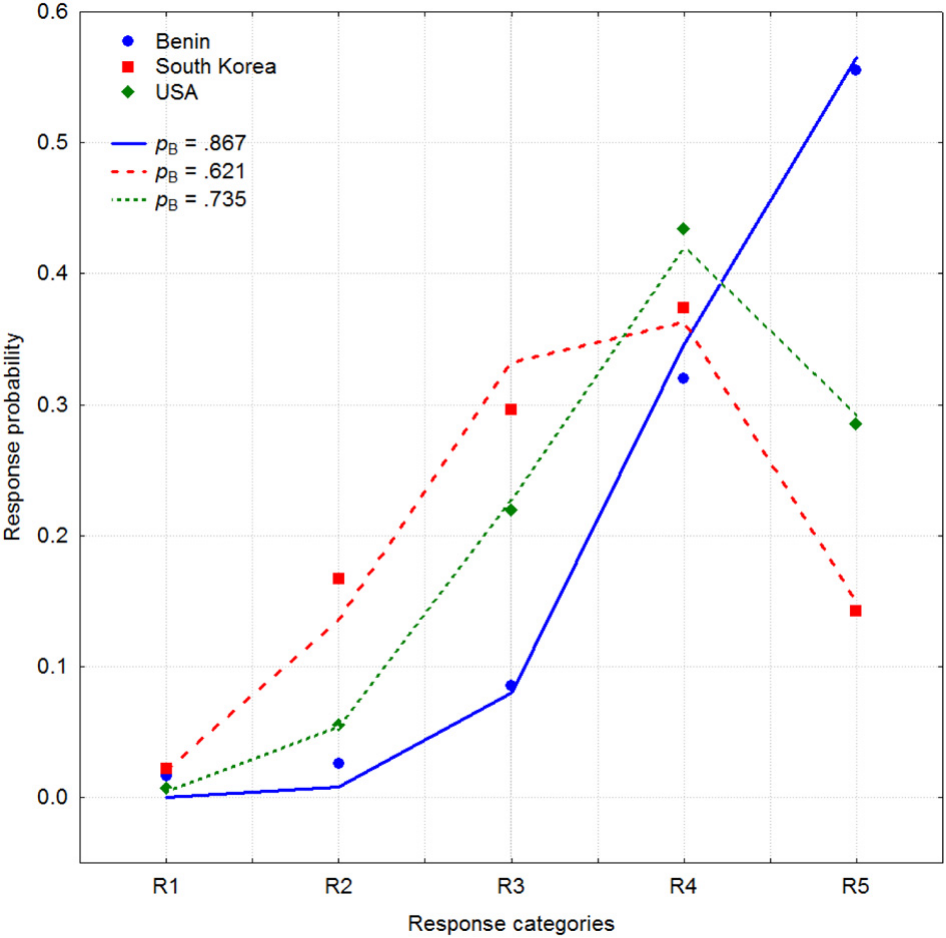
**Aggregated response frequencies on 6 Conscientiousness items for three countries: Benin (blue circles), South Korea (red squares), and the United States (green rhombus) (Mõttus et al., 2011)**. Three binomial distributions with values of parameter *p*_B_ = 0.867, 0.621, and 0.735 are shown by blue, red, and green lines.

Next I would like to present an example of a binomial function providing a good approximation to how answers are distributed on a Likert scale. For instance, in a recent cross-cultural study (Mõttus et al., 2012a), participants from 21 countries evaluated their own Conscientiousness on 6 NEO Personality Inventory facets Competence, Order, Dutifulness, Achievement Striving, Self-Discipline, and Deliberation using a bipolar rating scale with the negative side of the trait described on one end of the scale and the positive side on the other (Terracciano et al., 2005). For the Competence facet, for example, participants had to rate, on a five-point scale, their position between the end points of the trait defined as “capable, efficient, competent” and “inept, unprepared.” Figure 1 demonstrates response frequencies pooled across these six items and all participants for three different countries: Benin (blue circles), South Korea (red squares), and the United States (green rhombus). It is clear that, on average, Benin participants rated their conscientiousness higher than U.S. participants who, in turn, judged their own conscientiousness higher than South Koreans who were one of the lowest among the 21 nations.

These three distributions of answers are surprisingly similar to a binomial distribution b(*r*_i_, *p*_B_, *n*) with the probabilities *p*_B_ = 0.867, 0.621, and 0.731 respectively. Figure 1 demonstrates three binomial distributions shown by lines corresponding to these values which rather closely follow empirically observed response frequencies. It is perhaps even needless to say that approximation of the same data with a two-parameter normal CRC provides a relatively poor fit to these data.

The binomial distribution is the discrete probability distribution of the number of successes *r*_i_ = 0, 1, …, *n* in a sequence of *n* independent experiments each with two possible outcomes (“yes” or “no”), with a fixed success rate *p*_B_ across all these experiments:

(1) $$b\left(r_{i},p_{B},n\right)=\frac{n!}{r_{i}!(n-r_{i})!}p_{B}^{r_{i}}{\left(1-p_{B}\right)}^{n-r_{i}}$$

In order to separate the binomial probability *p*_B_ from the symbol of statistical significance a subscript “B” referring to binomial distribution is used. In confirmation of the visual impression, a Pearson chi-square (χ^2^) test (the sum of the squared differences between the observed and predicted frequencies, divided by the predicted frequency) showed that, in all three cases, the binomially expected frequencies do not differ significantly from the empirically observed response probabilities (*p* < 0.05). Consequently, the empirically observed response frequencies seem to be predicted solely by the parameter *p*_B_ in the binomial distribution b(*r*_i_, *p*_B_, 4). In the IRT terminology *p*_B_ corresponds to the item “difficulty” although in the personality domain it is pointless to describe, for example, extraversion more “difficult” than introversion or vice versa. To express the binomial function b(*r*_i_, *p*_B_, 4) in an explicit form, the probabilities *f*_1_, *f*_2_, … *f*_5_ that one of the five answers on a Likert-scale was chosen can be predicted by the following five equations:

(2) $$(\text{R}1)f_{5}={\left(1-p_{\text{B}}\right)}^{4}.$$

(3) $$(\text{R}2)f_{4}=4\cdot p_{\text{B}}\cdot {\left(1-p_{\text{B}}\right)}^{3},$$

(4) $$(\text{R}3)f_{3}=6\cdot p_{{\text{B}}^{\text{2}}}\cdot {\left(1-p_{\text{B}}\right)}^{2};$$

(5) $$(\text{R}4)f_{2}=4\cdot p_{{\text{B}}^{\text{3}}}\cdot (1-p_{\text{B}});$$

(6) $$(\text{R}5)f_{1}=p_{{\text{B}}^{\text{4}}};$$

If responses are close to these five predicted probabilities, as it is in Figure 1, then it is likely that these responses are related to a latent variable by a binomial process. It is relevant, however, to recognize that binomial distribution having only one parameter *p*_B_ is more restrictive than normal distribution determined by its mean and standard deviation. For example, on a 5-point Likert scale it is impossible that the middle response category R3 can be chosen with a probability higher than 0.375. Thus, any empirical distribution of answers in which the midpoint of the scale is chosen with higher frequency than 37.5% is incompatible with binomial distribution. Of course, there is no such restriction for normal distribution.

Up to now this narrative replicates almost exactly an excellent paper written by David Andrich who introduced a binomial latent trait model for the study of Likert-type items (Andrich, 1978). However, from this point onward the current exposition deviates from what was proposed by Andrich (1978). As a next step, he demonstrated how to transform binomial probability *p*_B_, with a double subscript corresponding to a single subject and a specific item, into the logistic form. In the particular case where *n* = 1 (responses are scored as either 0 or 1) the logistic transformation reduces to a Rasch's simple logistic model (Rasch, 1960). In other words, the binomial response model was transformed into generalized logistic model and because of it lost its self-sufficient meaning. Indeed, if a binomial distribution can be approximated and replaced either by logistic and normal functions then there is no need for extra assumptions beyond those of the already existing and well-known IRT models. This model was applied to 16 items asking how much children liked school. After eliminating 18 out of 309 fifth-year schoolchildren and 3 poorest fitting items a good agreement between the model and data was achieved (Andrich, 1978).

In this paper, however, I entertain an idea that a binomial process itself could be an adequate manifestation of a latent variable which in consequence determines distribution of answers on a Likert scale. One possibility for conceptualizing this binomial process is to imagine that respondents, before giving their answer to an item, execute a latent binomial mini-experiment. There is no need that the subject is aware of this mini-experiment or she or he is able to describe it introspectively. This hypothetical mini-experiment consists of four (one less than the number of response alternatives) mini-trials, each of which requires a simple indication whether or not the respondent agrees with the content of the posed question. The number of the successful items in this mini-experiment determines the answer chosen on the Likert-scale item. This binomial mini-experiment needs to satisfy the following four conditions:

1. There are four hidden mini-trials which together produce five possible outcomes;
2. Each mini-trial is independent of the others;
3. There are only two outcomes—“yes” or “no”—in each mini-trial, expressing endorsement of the core idea articulated in the item;
4. The probability of each outcome remains constant throughout all four mini-trials.

When these four conditions are fulfilled, the degree of endorsement of an item is described by the binomial function. Of course, the metaphor of a binomial mini-experiment is only one way to conceptualize this latent decision process. There are many other possibilities to describe the judgments that respondents make about their own or somebody else's personality traits when confronting a Likert-type scale. What is essential is for the binomial distribution response categories that were chosen to have truly numerical equivalents. For example, the choice response category R1 (“strongly disagree”) corresponds to 4 “mini-disagrees,” R2 (“disagree”) corresponds to 3 “mini-disagrees” and one “mini-agree” until R5 (“strongly agree”), which is the result of 4 “mini-agrees.” Since outcomes can be expressed in enumeration, this not only entails that the response categories are ordered R1 < R2 < R3 < R4 < R5, but that the intervals between two successive categories remain numerically the same across the whole Likert scale. Indeed, if the response distribution on a Likert scale item satisfies a binomial distribution, it is justified to operate with the responses as natural numbers. Since two and four are two and four mini-endorsements, respectively, it is absolutely justified to say that, in sum, there were 6 mini-endorsements or exactly 3 mini-endorsements per item. In short, the respondents seem to operate with the response categories as if they were natural numbers that can be counted, provided that responses obey binomial distribution.

Of course, not all empirically observed response distributions on a Likert-type scale obey a binomial distribution. From a statistical point of view, it is highly unlikely that all response probabilities are strictly related to one another by a set of restrictive formulas depending on only one free parameter—the probability of endorsing a latent statement. As a confirmation of this observation, many actual response distributions in the above mentioned study depart considerably from the binomial law (Mõttus et al., 2012a). However, to the best of my knowledge, in very few studies binomial function was used as a response function which connects latent states to explicit responses on a Likert scale item (cf. Andrich, 1978). In some other applications binomial function was used to describe sampling or some other processes that are relevant for the IRT (Lord and Novick, 1968; van der Linden, 1979; Gross and Shulman, 1980).

One of the main goals of this paper is to develop a binomial IRT model which can be used as a benchmark relative to which better known two-parameter normal or logistic IRT models can be evaluated. In this study I am not intending to study all 30 subscales of the NEO Personality Inventory each of which consists of eight items. More modest goal is to observe how the distribution of answers on each 240 Likert-type items can be fitted in isolation with a binomial function. It is very unlikely that one specific IRT model is superior of others in majority of conditions. More realistic is to elucidate not very well understood characteristics of data which make them better or worse harmonizing with different response functions.

Beside this central goal there are some other problems to solve one of which is related to a well-known fact that Likert scales are vulnerable to several distortions. Respondents may have, for instance, a tendency to use extreme categories (extreme response style) more often than could be expected from purely statistical considerations (McGrath et al., 2010; Mõttus et al., 2012b). Likewise, some other group of respondents may have an inclination to use a middle category (“neutral,” “not sure,” “undecided”) more frequently than could normally be expected (Dubois and Burns, 1976; Hernandez et al., 2004; Kulas and Stachowski, 2009). Although operationally it is easy to define the extreme response style as a frequency of using extreme response categories (“strongly agree” and “strongly disagree”), it is tremendously difficult to separate the “normal” use of any of these response categories from their disproportionate or biased use. In other words, my proposal is to use a binomial response model as a reference relative to which biases can be consistently defined. Deviations from the expected theoretical distribution are natural candidates in the search for response biases.

One reason why not all empirically observed response distributions on a Likert-type scale follow a binomial distribution may be that not all respondents understand and answer items identically. Constructors of personality questionnaires mostly take for granted that all respondents use the response categories to an item in basically the same way. In many cases, this is nothing but good faith because until recently there were no versatile tools to identify different subgroups of respondents who interpret response categories differently. Fortunately, several mixed-measurement or latent class models were developed to identify two or more subgroups of respondents who interpret response categories in a different way (Rost, 1990, 1991; Bolt et al., 2001; Asparouhov and Muthen, 2008; Maij-de Meij et al., 2008; Muthen and Asparouhov, 2009). A bad fit to a binomial response model may be an indicator of at least two groups whose response patterns can be described by two different binomial response models. One of the main goals of this paper is to test how much a simple binomial response model can be amended by a mixture-binomial model which assumes two different subpopulations that have two different probabilities *p*_B_ with which they endorse a given item.

## Methods

Participants came from the Estonian Genome Center (EGC) bio bank, which is currently affiliated with the University of Tartu (for details see www.biobank.ee). The EGC participants have been randomly selected from individuals visiting general practitioners (GP) and hospitals and recruited by GPs and hospital physicians. All participants have provided informed consent. In addition to donating blood samples and answering a medical questionnaire, participants were asked to complete the self-report Estonian version of the NEO Personality Inventory-3 (NEO PI-3; McCrae et al., 2005) and also find a knowledgeable informant who would complete the same questionnaire about them. The NEO PI-3 is a slightly modified version of the NEO PI-R questionnaire (Costa and McCrae, 1992) that was adapted into Estonian by Kallasmaa et al. (2000). Like its predecessor the NEO PI-R, the NEO PI-3 has 240 items which measure 30 personality traits grouped into the five personality domains: Neuroticism (N), Extraversion (E), Openness (O), Agreeableness (A), and Conscientiousness (C). Each domain is represented by 6 facets. For example, Neuroticism is measured by N1: Anxiety, N2: Angry Hostility, N3: Depression, N4: Self-Consciousness, N5: Impulsiveness, and N6: Vulnerability. Each facet is measured by 8 items, approximately a half of which are keyed in the opposite direction. Participants were asked to express their agreement or disagreement with the content of each item on a 5-point scale with the following response categories: 1 = strongly disagree, 2 = disagree; 3 = neutral; 4 = agree, and 5 = strongly agree. Some of these personality data have been used in other papers (e.g., Allik et al., 2010b; de Moor et al., 2012; Mõttus et al., 2012c,d).

In total, the sample used in the present study included 1731 people (of whom 991, or 57.3%, were women) with a mean age of 42.8 years (*SD* = 16.5, ranging from 18 to 89 years). Informants were slightly younger than their targets with a mean age 39.9 years (*SD* = 15.5, ranging from 12 to 89). Of the informants, 52% were spouses or partners, 15% friends, 12% parents, 9% children or grandchildren, 6% siblings, 3% acquaintances, and 3% were categorized as other relatives. Women comprised 68.9% of the informants.

## Results

### Comparison of one-parameter binomial (1PBM) and two-parameter normal (2PNM) models

**Figure 2:**
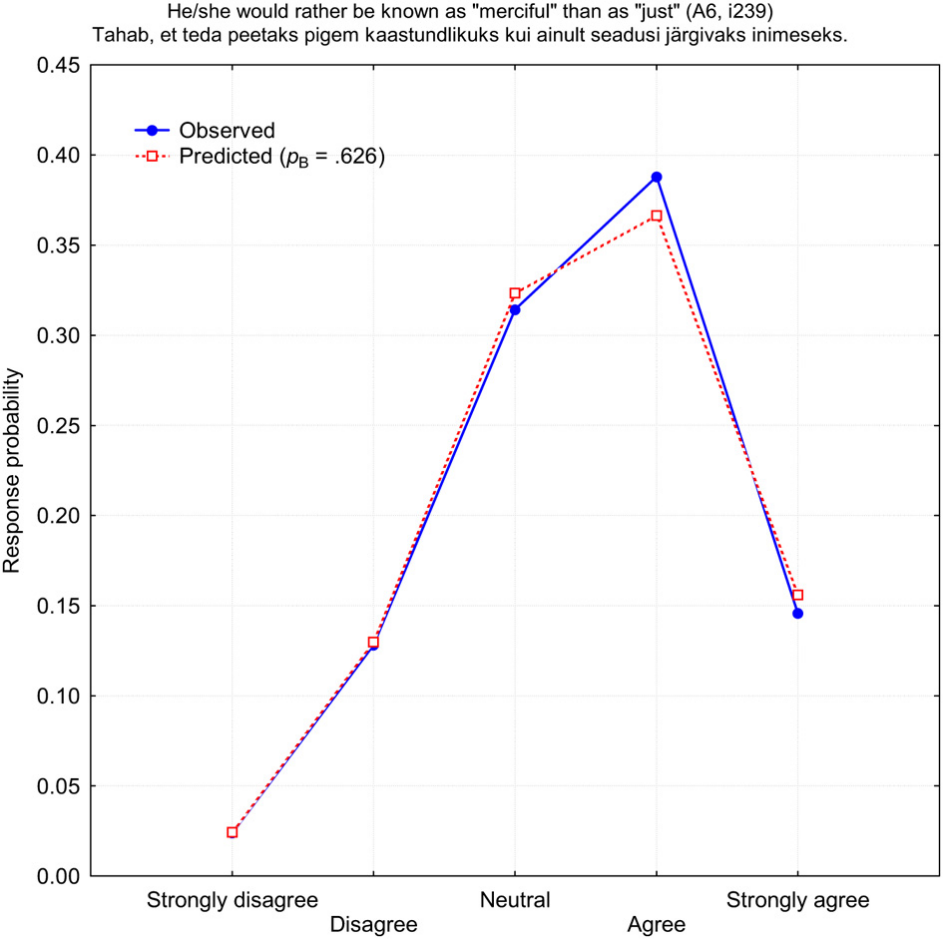
**Observed (blue) and predicted (red) response probabilities for the other-rated item *i*239 (A6: Tender-Mindedness)**.

To begin with, I searched for the binomial function b(*r*, *p*_B_, 4) which could best predict response frequencies on all 240 NEO PI-3 items for both self- and observer-ratings. In terms of the IRT classification, this is one-parameter binomial model (1PBM) applied to each item in isolation. The program looked through all possible *p*_B_-values and selected the one which provided the best fit to the observed response frequencies in terms of the minimal χ^2^-value. Since χ^2^-statistics depend directly on the number of observations *N* = 1731, it was unrealistic to expect a generally good statistical fit. Indeed, only one other-rated item *i*239 “He/she would rather be known as ‘merciful’ than as ‘just”’ had a response distribution sufficiently close to a binomial function at the level of 5% significance: χ^2^_(4)_ = 5.668, *p* = 0.225. Figure 2 demonstrates the distribution of observed and predicted response frequencies for the item *i*239. All other items demonstrated poor fit to a binomial distribution, in terms of the χ^2^-statistics, at least.

It is important to stress that there was no substantial loss of information in computing the *p*_B_-value for each item, compared to a more customary arithmetic mean of all responses. The mean scores of all 240 NEO PI-3 items were extremely highly correlated with the computed binomial probabilities *p*_B_, identically for self- and other-ratings (*r* = 0.998, *p* < 0.000001).

Next, for a comparison, I fitted two-parameter normal model (2PNM) to the same set of data. In spite of an additional parameter 2PMN approximation was not palpably superior of the 1PBM approximation. Out of 480 response patterns not a single χ^2^-value was below 9.4877 (5% significance level for four degrees of freedom). Also both the Kolmogorov-Smirnov and Shapiro-Wilk tests rejected the hypothesis that the observed distributions of responses were normal in each and every case by a considerable margin.

**Figure 3:**
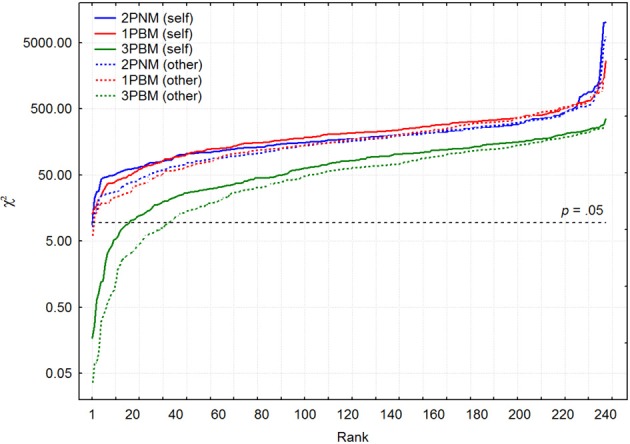
**The patterns of χ^2^ growth on a logarithmic scale for 1PBM, 2PNM, and 3PBM separately for self (continuous lines) and other (broken lines) ratings**.

In order to compare fits of these two models, 1PBM and 2PNM, χ^2^-values for self- and other-ratings were arranged into increasing order. Figure 3 depicts the pattern of χ^2^ growth for these two models for both self- and other-ratings.

Generally speaking, there is no substantial difference between one-parameter binomial and two-parameter normal approximation. It seems, however, that other ratings are slightly better approximated by both binomial and normal models. The median χ^2^-values were 173.9 and 215.6 for 2PNM and 1PBM respectively for self-ratings. For other ratings these χ^2^-values were slightly lower: 160.2 and 168.2 respectively. It is important to mention that using some other goodness of fit criteria that could account for differences in the number of parameters (e.g., Akaike Information Criterion which in the current case is *AIC* = χ^2^ + 2*k*, where *k* is the number of parameters) was not able to demonstrate indisputable advantage of either 1PBM or 2PNM models.

### Estimation of possible noise

**Figure 4:**
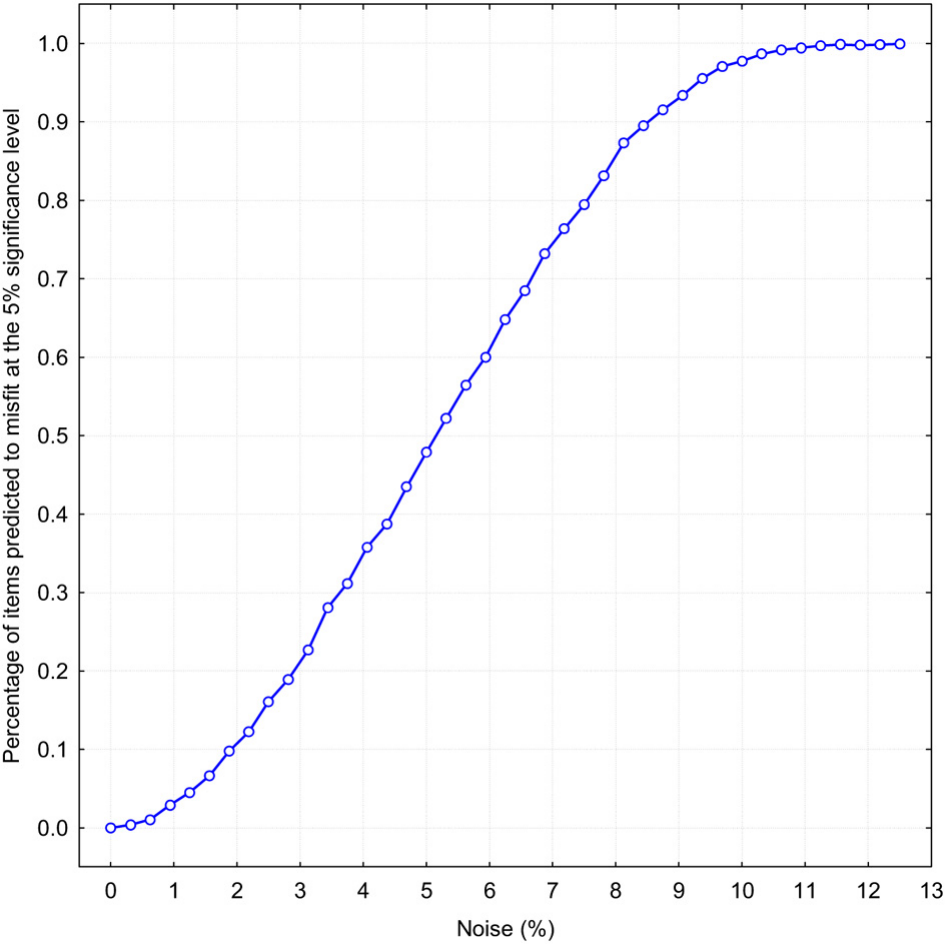
**Proportion of items predicted to misfit at the 5% significance level on χ^2^-statistics as a function of random noise percentage**.

Because the chi-square statistic is very stringent, especially at a large number of observations, more realistic measures should be considered. One promising idea is to determine how resistant the binomial fit is to the addition of random noise. The basic idea is to add a different percentage of random noise to a perfect binomial distribution for establishing how much noise can be tolerated before binomial approximation starts to deteriorate. If all *N* = 1731 responses on a single item are distributed exactly according to the binomial function b(*r*_i_, *p*_B_, 4), then the level of noise is evidently zero and there will be no approximation error. Let suppose that not all 100% of responses are distributed binomially, but only a fraction of them. The remaining percentage of answers *K* will be distributed randomly, so that the occurrence of each response alternative *R*_i_ has an equal probability. Thus the ratio 100 · *K/N* determines the percentage of noise. By increasing the percentage of noise we can observe how the average misfit increases with the added noise. In order to make such a simulation maximally realistic, I selected 240 previously obtained binomial *p*_B_-values which were used to approximate self-ratings with a binomial function (the mean *p*_B_-value was 0.56 with *SD* = 0.13). Figure 4 demonstrates how the binomial fit tolerates addition of different amounts of random noise. The abscissa is the probability that the obtained χ^2^-statistic exceeds the 5% significance level, which, for four degrees of freedom, is χ^2^ = 9.4877. This figure tells us that slightly more than 5% noise makes already half of the NEO PI-R 240 items misfit to a binomial distribution, even if 95% of answers were given according to a perfect binomial law. If there is 12% of noise, then it would be totally unrealistic to expect that any of the 240 items has a statistically significant agreement with a theoretical prediction based on the binomial function. It is important to remember that if we divide this 12% noise equally between five possible response categories then already 2.4% deviation from the expected frequency would be an intolerable imprecision. It is perhaps worth mentioning that typical estimates of measurement error in personality research based on Likert-type scales are usually estimated higher than 12% (Viswesvaran and Ones, 2000; McCrae et al., 2001, 2011). On the whole, this hypothetical level of noise can be used as a more realistic estimate of the approximation quality of the binomial function.

Strictly speaking this modeling result means that by adding one additional parameter—random noise—to 1PBM it is possible to make a binomial model entirely fit with the observed response patterns. What is particularly noteworthy it is sufficient that only in a limited number of trials the attention of respondents is distracted and they respond erratically to a relatively small number of items.

### Mixed binomial model (3PBM)

**Figure 5:**
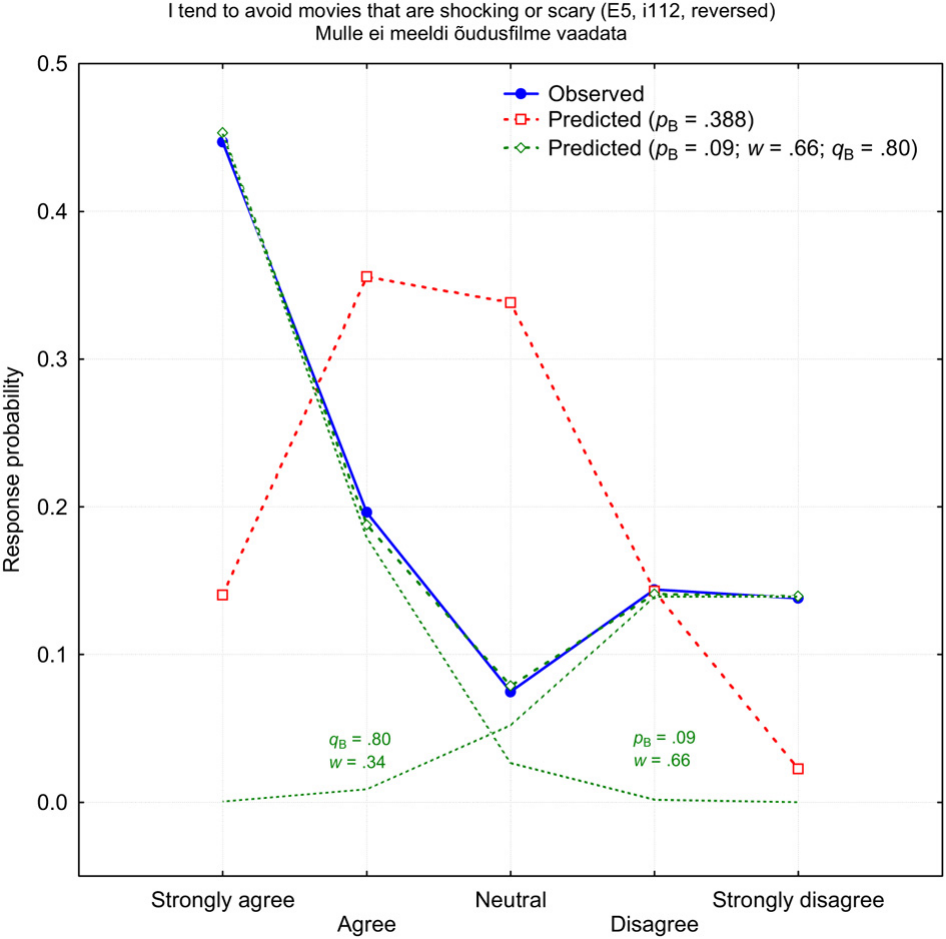
**Observed (blue) and predicted (red) response probabilities for the self-rated item *i*112 (E5: Excitement-Seeking)**. The green shows a mixed-binomial approximation with two differently weighted binomial components *p*_B_ and *q*_B_.

There were few items which response patterns could be fitted with neither binomial nor normal function in principle. These response patterns typically have two maximums and the minimum in the middle of the response scale (V-shape). The worst prediction was for the self-rated item *i*112 “I tend to avoid movies that are shocking or scary,” which measures E5: Excitement Seeking. Figure 5 demonstrates the observed response probabilities (blue) and the best fitting binomial prediction (red). Please ignore the green lines and symbols for the time being. Obviously, the 1PBM cannot provide a reasonably good fit to an empirical distribution which has such a distinctive V-shape: χ^2^_(4)_ = 2654.1, *p* < 0.000001. As expected, these two more or less polarized groups who either like or dislike scary movies are distinctive by demographic variables. These were dominantly women and participants with higher education who disliked horror movies. Among those who enjoyed shocking experiences were a disproportionally large number of men who had only elementary education. It cannot be excluded that some of participants ignored the negative form “don't like” in Estonian (“ei meeldi”) and answered as if it were an affirmative item.

It is likely that different groups of participants use and interpret Likert items in different ways. A characteristic V-shape may be suggestion to use a mixed binomial model instead of 1PBM which seems to be unable to explain this type of response pattern. Technically, it means that different groups rely on different binomial processes, which are governed by two different binomial parameters, *p*_B_ and *q*_B_. While partitioning normally distributed variables into different subgroups is not an easy task (e.g., Lahti et al., 2002), it is certainly less problematic with a binomial distribution which depends only on one parameter rather than two, as is the case with a normal distribution.

A program was written to search for the best fit for the following mixed-binomial functions:

(7) $$f_{\text{i}}=w\cdot b(r_{\text{i}},p_{\text{B}},4)+(1-w)\cdot b(r_{\text{i}},q_{\text{B}},4),$$

where *f*_i_ is the expected response probability of the i-th response alternative (i = 0, 1, …, 4), *p*_B_ and *q*_B_ are two different binomial probabilities, and *w* is the weight with which the first of the two binomial components is taken. Since there were 3 parameters in the model we can call it three-parameter binomial model (3PBM). The best fit was searched for with an exhaustive method among all possible combinations of *p*_B_, *q*_B_, and *w*.

A significant improvement can be defined as a reduction in the χ^2^-statistic for a mixed-binomial model or 3PBM of at least 5%, compared with a single binomial model fit. According to this criterion, there were 25 (10.4%) self-rated and 31 (12.9%) other-rated items which failed to show any improvement by applying a mixed-binomial model. The ranked distribution of χ^2^-values for self-rated and other-rated items is shown in Figure 3. The goodness of fit has considerably improved relative to both 1PBM and 2PNM. As the quality of the approximation improved, 18 (7.5%) self-rated and 36 (15%) other-rated items achieved agreement between the observed and predicted frequencies at a 5% significance level. On average, the χ^2^-statistics improved by 52% for both self- and other-ratings. In spite of this substantial improvement, the median χ^2^-statistics remained clearly above the critical 5% significance level: 81.7 and 62.4 for self- and other-ratings, respectively. This improvement is not surprising considering the fact that a quite large number of response distributions demonstrated V-pattern at least partially. For example, the number of response patterns for which the response frequency to the middle category R3 was smaller than to the neighbouring response categories R2 and R4 simultaneously was unexpectedly high: 45 and 34% out of all 240 items had a V-shape in the middle of the scale for the self- and other-ratings respectively. Thus, there was persistent tendency to use a middle category (“neutral” or R3) less frequently than could be normally expected. Since neither binomial nor normal distributions can have a local minimum in the middle of the scale it is not surprising that simple response models 1PBM and 2PNM did not have a good fit to data.

Unfortunately, there is no possibility to compare 3PBM performance with a mixed normal model. In addition to two normal distributions with their means and standard deviations it is also necessary to specify weight *w* with which the first of the two normal components is taken. In the result the number of model's parameters equals to the number of response categories which makes approximation meaningless: the number of model's parameters is equal to the number of observed variables.

It is interesting to notice that the computed χ^2^ mean values for self- and other-ratings correspond to about 13 and 11% noise levels, respectively, which were determined by the above described simulation procedure. These two estimates are slightly higher than that which could be deduced from Figure 3. Considering the number of items which passed or failed to pass the χ^2^ significance test at the 5% level, the estimates of the noise percentages are closer to 8 and 9% for other- and self-ratings, respectively.

There is no space to reproduce approximation statistics for all 240 NEO PI-3 self- and other-rated items. For reasons of economy, only the 20 best and worst items for both self-ratings and other-ratings are reproduced in Table 1.

**Table 1:** **Twenty best and worst NEO PI3 self- and other-rated items in terms of the χ^2^-statistic for a mixed-binomial approximation**.

	**Self-ratings**							**Other-ratings**						
**Rnk**	**Item**	**Subscale**	***p*_B_**	***q*_B_**	***w***	**χ^2^**	**Impr%**	**Item**	**Subscale**	***p*_B_**	***q*_B_**	***w***	**χ^2^**	**Impr%**
1	*i*23	O5	0.26	0.67	0.52	0.17	99.9	*i*117	E6	0.48	0.78	0.71	0.03	99.9
2	*i*68	O2	0.71	0.12	0.51	0.26	100.0	*i*11	N3	0.52	0.25	0.72	0.07	99.8
3	*i*13	O3	0.78	0.43	0.62	0.63	99.6	*i*74	A33	0.81	0.39	0.66	0.07	100.0
4	*i*129^*^	A2	0.86	0.26	0.94	0.82	99.8	*i*38	O2	0.83	0.40	0.60	0.11	100.0
5	*i*58^*^	O6	0.83	0.30	0.97	1.19	98.2	*i*14	A3	0.85	0.38	0.65	0.30	99.9
6	*i*112	E5	0.09	0.80	0.66	1.26	100.0	*i*30	C6	0.69	0.22	0.72	0.38	99.9
7	*i*53	O5	0.69	0.19	0.67	2.29	99.6	*i*69	A2	0.71	0.37	0.61	0.45	99.7
8	*i*72^*^	E3	0.66	0.27	0.52	3.25	98.6	*i*123	O1	0.44	0.84	0.87	0.60	99.1
9	*i*3	O1	0.79	0.52	0.65	3.82	93.9	*i*27	E6	0.73	0.36	0.80	0.69	99.3
10	*i*98	O2	0.73	0.31	0.60	4.25	98.5	*i*72^*^	E3	0.36	0.70	0.53	0.84	99.4
11	*i*239	A6	0.68	0.40	0.79	5.25	85.9	*i*130	C2	0.87	0.34	0.93	0.91	99.7
12	*i*234	A5	0.64	0.98	0.96	5.43	58.0	*i*129^*^	A2	0.82	0.26	0.95	1.20	99.3
13	*i*89	A6	0.76	0.36	0.85	5.87	94.9	*i*211	N1	0.57	0.27	0.72	1.88	96.8
14	*i*92	E1	0.60	0.01	0.99	7.19	52.5	*i*158^*^	O2	0.80	0.35	0.72	2.21	99.4
15	*i*170	C4	0.74	0.52	0.66	7.56	73.5	*i*113	O5	0.63	0.28	0.53	2.67	98.2
16	*i*118	O6	0.86	0.36	0.98	8.21	74.4	*i*203	O5	0.62	0.40	0.57	2.76	88.4
17	*i*218	O2	0.23	0.77	0.91	8.91	95.7	*i*208	O6	0.60	0.34	0.53	3.03	93.2
18	*i*28^*^	O6	0.73	0.41	0.53	9.14	93.0	*i*50	C4	0.77	0.44	0.83	3.26	94.7
19	*i*84	A5	0.52	0.98	0.95	10.26	73.2	*i*128^*^	O2	0.62	0.17	0.58	3.31	99.2
20	*i*186	N2	0.38	0.97	0.98	10.46	56.8	*i*20	C4	0.76	0.39	0.61	3.50	98.2
220	*i*194^*^	A3	0.72			202.62	0.0	*i*216	N2	0.74	0.29	0.63	187.27	59.9
221	*i*166	N4	0.29	0.64	0.70	207.90	25.9	*i*109	A4	0.65	0.29	0.56	188.36	38.8
222	*i*115	C5	0.66	0.33	0.72	216.70	19.6	*i*151	N1	0.35	0.72	0.56	189.55	41.4
223	*i*90^*^	C6	0.28	0.64	0.75	218.04	24.1	i180	C6	0.69	0.35	0.83	193.70	15.4
224	*i*167^*^	E4	0.72	0.36	0.66	218.50	30.4	i236	N6	0.28	0.66	0.67	193.81	38.1
225	*i*205	C5	0.30	0.63	0.65	220.59	23.2	*i*220^*^	C2	0.70	0.23	0.55	199.29	64.2
226	*i*190^*^	C2	0.26	0.75	0.63	221.48	64.3	*i*90^*^	C6	0.33	0.70	0.61	200.69	36.9
227	*i*206^*^	N6	0.34			222.26	0.0	*i*81	N5	0.30	0.65	0.67	206.42	28.1
228	*i*228	O4	0.30	0.68	0.74	225.20	28.9	*i*156	N2	0.64	0.31	0.61	213.01	24.1
229	*i*154	A1	0.63	0.31	0.78	228.89	14.0	*i*237	E6	0.71	0.36	0.77	215.77	19.6
230	*i*85	C5	0.70	0.22	0.96	231.48	8.5	*i*167^*^	E4	0.73	0.37	0.66	215.81	29.5
231	*i*56	N6	0.22			234.91	0.0	*i*206^*^	N6	0.30			219.26	0.0
232	*i*225	C3	0.75	0.28	0.85	242.37	39.1	*i*176	N6	0.26			220.21	0.0
233	*i*229^*^	A4	0.26	0.69	0.93	250.80	10.2	*i*183	O1	0.54			231.26	0.0
234	*i*104	A3	0.80			256.28	0.0	*i*214	A1	0.71			241.74	0.0
235	*i*220^*^	C2	0.69	0.23	0.57	257.56	54.9	*i*194^*^	A3	0.73			242.72	0.0
236	*i*215	C1	0.75			258.36	0.0	*i*229^*^	A4	0.26	0.67	0.85	244.36	23.5
237	*i*164	A3	0.63			262.50	0.0	*i*231	N5	0.62	0.32	0.51	254.81	16.4
238	*i*126	N2	0.35	0.80	0.93	282.19	11.6	*i*121	N1	0.33	0.63	0.56	255.79	16.6
239	*i*65	C1	0.73			286.76	0.0	*i*178	O6	0.71	0.31	0.94	263.09	6.3
240	*i*134	A3	0.56			353.82	0.0	*i*190^*^	C2	0.25	0.78	0.66	269.31	68.3

Imp%, the percentage of improvement of a mixed-binomial approximation (3PBM) relative to a single binomial approximation (1PBM);

* *Items which appear in both parts of the list in self- and other-ratings. Missing q_B_ and w values mean that a mixed-binomial function improved the approximation less than 5%*.

Many items appear in both sides of these lists of self- and other-ratings. This is not surprising because the mean values of self-ratings are highly correlated with the mean values of other-ratings over all the 240 NEO PI-R items (*r* = 0.90, *p* < 0.00001). The correlation between the χ^2^-statistics (*r* = 0.62, *p* < 0.00001) also indicates that many items which are either good or bad in terms of a mixed-binomial approximation are sufficiently similar for self- and other-ratings. However, it is also evident that other-ratings obey a binomial distribution slightly more precisely than self-ratings.

**Figure 6:**
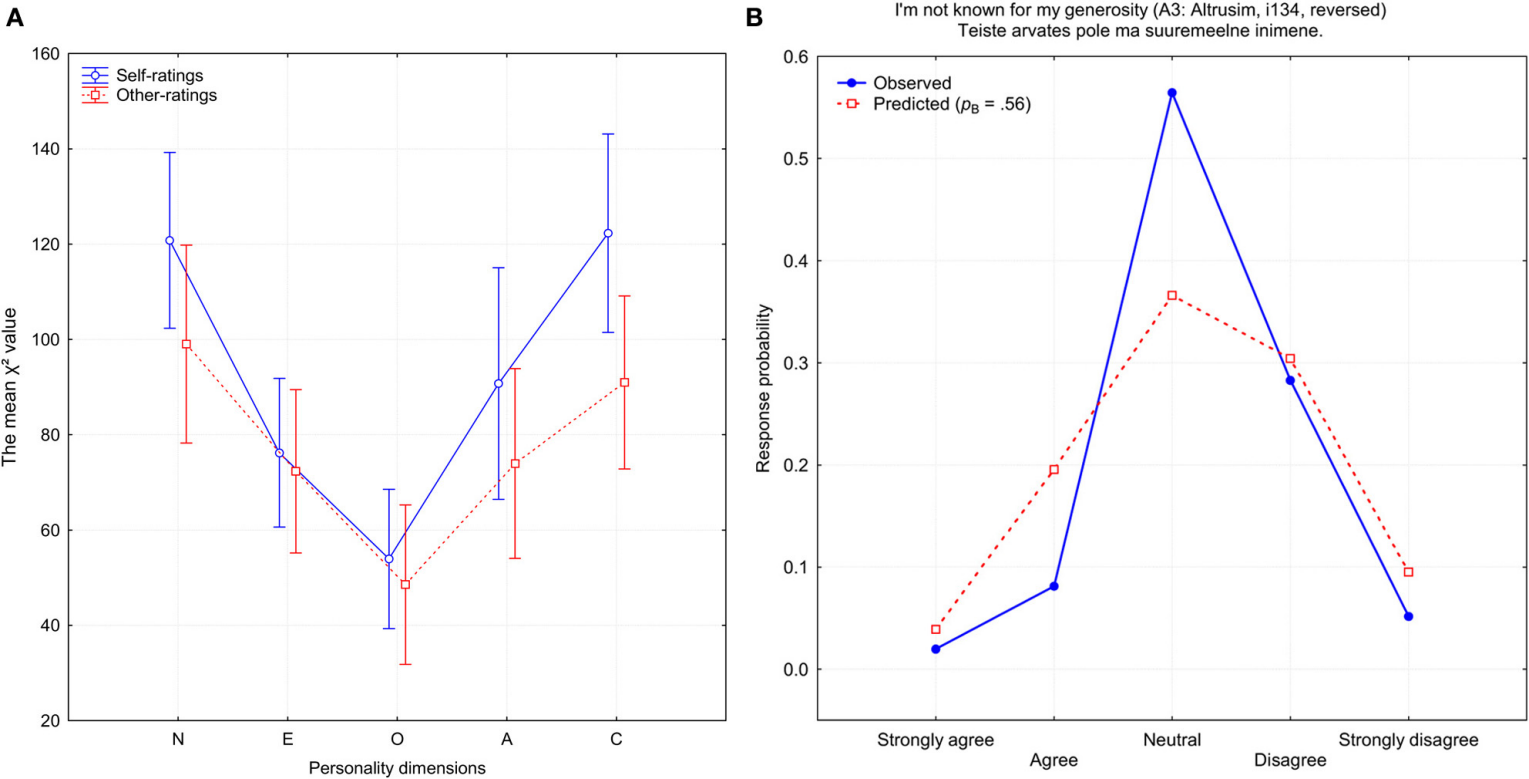
**(A)** The mean χ^2^ approximation error for items belonging to one of the five personality dimensions: N, Neuroticism; E, Extraversion; O, Openness, A, Agreeableness, and C, Conscientiousness. **(B)** Observed (blue) and predicted (red) response probabilities for the self-rated item *i*134 (A3: Altruism).

How well an item was approximated by a mixed-binomial model also depended on which personality dimension it was supposed to measure. Figure 6 demonstrates the mean χ^2^ approximation of errors for items belonging to one of the five personality dimensions. Items which were created to measure Openness demonstrated the best fit with the mixed-binomial distribution. In both of the categories of ratings, self-ratings and observer-ratings, the most problematic were the Neuroticism and Conscientiousness items, which exhibited on average the worst fit to a mixed binomial distribution. Interestingly, when I computed the mean number of potentially biased items, the general shape replicated the pattern shown in Figure 6. A biased item was defined as one which had at least one response category on which theoretically expected and empirically observed frequencies differed by at least 10% (there were 56 self-rated and 41 other-rated items which were biased).

One possibility is that the χ^2^ approximation error is somehow related to the content of the items. For example, items with higher or lower social desirability content may demonstrate a different fit to the theoretical model. In order to study this possibility, I computed the mean χ^2^ approximation error for each of the 30 NEO PI-R subscales and compared these mean values with previously reported social desirability ratings for the same subscales (Konstabel et al., 2006). Approximation errors did not correlate significantly with social desirability ratings (*r* = −0.09, *p* = 0.623 and *r* = −0.17, *p* = 0.363 for self- and other-ratings, respectively). No evidenced was found that the approximation error is related to the visibility of personality traits (cf. Allik et al., 2010b) or from whose perspective they are judged (Allik et al., 2010a).

One telling example for the dramatic improvement after a mixed-binomial approximation is the above mentioned item *i*112 (Figure 6). The worst item in a single binomial prediction turned into an exemplary item in a mixed-binomial prediction: χ^2^_(4)_ = 1.255, *p* = 0.868. It is very likely that a characteristic V-shape is determined by two disparate binomial functions with the parameters *p*_B_ = 0.08 and *q*_B_ = 0.80, respectively. Since the weight of the first function *w* = 0.66 is about two times larger than the weight of the second function (1 − w) = 0.34, the subgroup of those who tended to disagree was about twice more populous than the subgroup whose members were inclined to agree with the statement. Fascinatingly, a mixed-binomial approximation of item *i*112 was also successful for other-ratings [χ^2^_(4)_ = 4.834, *p* = 0.695], with parameters quite similar to those of self-ratings: *p*_B_ = 0.14, *q*_B_ = 0.73, and *w* = 0.55.

### Detecting response biases

However, the majority of items failed to reach a significant fit, even after applying a mixed-binomial model. Besides the constraints of the χ^2^ distribution themselves, the binomial distribution, as was already mentioned above, is a very tight distribution. For example, it excludes the possibility that the middle neutral category *R*3 could be chosen with a higher probability than 0.375. This limitation is clearly seen in the item *i*134 “I'm not known for my generosity” (reversed), which is shown in Figure 5. Since a mixed-binomial prediction failed to improve upon a single binomial prediction, only the latter is demonstrated. The main reason for the extremely bad approximation [χ^2^_(4)_ = 353.8, *p* < 0.0001] is the much higher than expected exploitation of the neutral category. There was no chance that a binomial function could have explained the 56.4% with which the neutral response was chosen.

**Figure 7:**
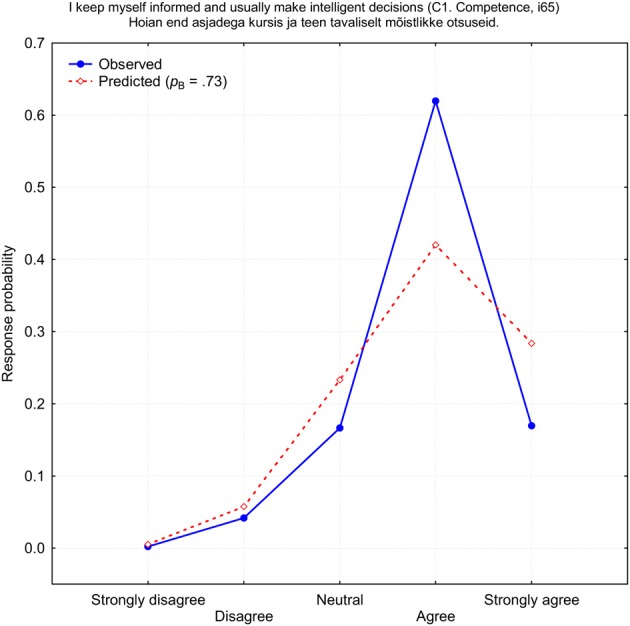
**Observed (blue) and predicted (red) response probabilities of the self-rated item *i*65 (C1: Competence)**.

This example demonstrates that if there is a bias toward one response category, where this is preferred more or less frequently than is expected by a binomial distribution, then it is unlikely that even a mixed-binomial model can provide a reasonably good fit to the observed response frequencies. On the other hand, deviations from the expected binomial distribution can be used as a criterion for a more precise definition of biases. Thus, bias is not every choice of response category, but only when such a choice has a sample level probability that is considerably higher or lower compared to theoretical expectation. Usually, researchers talk mainly about the frequencies with which the middle (neutral) or extreme response categories are used. However, not only the middle or extreme response alternatives can demonstrate bias. There are several items which apparently demonstrate a new type of bias which could be called a “weak agree” or “weak disagree” category bias. One of these items is shown in Figure 7. Like the previous example, this item, “I keep myself informed and usually make intelligent decisions” (*i*65), failed to improve after the mixed-binomial approximation was applied. Again, there was no chance that a binomial function can even reach close to the 62% of cases with which the answer “Agree” was chosen (a theoretical maximum is only 42.2%). For this reason, item *i*65 demonstrated the second worst fit: χ^2^_(4)_ = 186.8, *p* < 0.0001. It seems as if a considerable number of respondents were too shy to admit that they are well informed and make intelligent decisions. It seems that, for them, it was socially more desirable to support this statement moderately, not strongly, which could violate a socially desirable level of modesty.

There is no chance that strongly biased items can be tolerably approximated by either binomial or any continuous response functions. Also, there seems to be no simple rule which could describe why respondents start to use one of the response categories more or less frequently than it could be customarily expected on the basis of some model. This means that the best but not necessarily elegant solution would be an introduction of a certain number of *ad hoc* variables which would cover large deviations from the expected response frequencies.

## Discussion

It was remarkable that binomial distribution with a single parameter provided generally not significantly worse fit compared with two-parameter normal response curve. Although by formal fit values it was indecisive which of these two models is superior the single-parameter binomial model has several conceptual advantages:

1. The one-parameter binomial model, 1PBM, has only single parameter to be determined which is a considerable advantage before at least two parameters of the normal or logistic models;
2. Binomial is a discrete probability distribution assuming a limited number of latent events which is in a sharp contrast with normal and logistic models both of which are presuming an infinite continuum of latent states;
3. Although there has been a tremendous progress in the computing power which changes our perspective on the computational difficulty it is still much easier to determine values of a binomial function than to compute normal or even logistic functions;
4. The only parameter of binomial function probability *p*_B_ has more transparent interpretation than the “difficulty” parameter in normal and logistic models^1^. Unlike other IRT parameters binomial probability is dimensionless and completely independent of the number of response alternatives on the Likert scale.

The conception of latent variable in the form of a real numbered continuum seems so simple and intuitively irresistible that no one ever really contested it. Even though some thoughts occasionally went on more simple discrete probability distributions they were immediately brought back to normal or logistic functions (Andrich, 1978). In fact, nothing but habits and preconceptions prevents us from assuming that traits are represented not by an infinite set of events but rather by a finite set of psychological states. Of course, the idea of a latent performance of a series of mini-trials may seem initially extravagant. However, before reaching a premature verdict it is relevant to remember that the idea of latent mini-trials is nothing but a metaphor which primary function is to emphasize basic properties of an underlying process. There is nothing weird, for instance, in the assumption that personality traits such as feeling sympathy and concern for others—Tender-Mindedness—are represented by a set of semantic primes which are silently tested when an answer is searched for a given personality item (cf., Wierzbicka, 1996).

Without doubt, the strength of IRT consists not only in selecting a response curve to fit distribution of responses on an isolated item. A real power stems from a simultaneous analysis of multiple items related to a hypothetical underlying latent variable (van der Linden and Hambleton, 1997; Reise and Waller, 2009). However, the choice of the particular response function connecting latent trait to manifest responses has obvious consequences for some other inferences made on the basis of the IRT which could lead to misleading results (Meijer and Baneke, 2004). For example, it is interesting to discuss what would happen to the problem of measurement invariance provided that binomial response function proved to be a correct one in a given situation. Measurement invariance is a property of measurement that indicates that the same concept is measured across different groups. For example, before comparing scores collected in two different cultures, it is useful to verify that a given measure is interpreted in a conceptually similar manner by respondents representing these two cultures (e.g., Nye et al., 2008). There are different levels of measurement invariance (e.g., configural, weak, and strong) which specify the type of comparison permissible between these measures (Vandenberg and Lance, 2000). In principle, we cannot even compare self-ratings with observer-ratings before measurement invariance is established on one of these possible levels between these two measures. It would be a relatively easy call if, for example, self-ratings were represented by two sufficiently large subpopulations but other-ratings needed only one sample to approximate all data. Quite obviously, in this case, self- and other-rated data are not fully comparable because two completely different functions are needed to approximate responses. The decision becomes much more uncertain when the estimated number of subpopulations is identical. There seems to be no simple rule how to decide if two mixed-binomial distributions are identical. Since a binomial distribution has only one parameter, it is not entirely clear what to do with weak (metric) and strong (scalar) invariance, which are based on the understanding that a latent variable is related to manifest responses through an equation containing two parameters: the regression coefficient and residual constant. Without doubt, the binomial measurement model provides not only promise but also questions for future studies of measurement invariance.

There is a typical way how researchers react to a situation in which a proposed theoretical model does not fit empirical data very well (cf., Goldstein and Blinkhorn, 1982). For the start they usually recommend eliminating a considerable number of deviant respondents. Then they suggest excluding few items which may look abnormal and finally they may even advise to dismiss certain response categories (such as “neutral”) which are inflating the fit characteristics (cf., Andrich, 1978). Alternative and more productive strategy is to modify response models is such way that they could explain at least some of the observed anomalies. Results of this study made it absolutely clear that the binomial response curve has chances to describe accurately answers on a Likert scale only with one of two amendments based on diametrically opposite principles. The first of these two principles is random or erratic responding. As a simulation study demonstrated even a small amount of random answers could distort perfectly binomial response pattern to such an extent that according to a very strict χ^2^ criterion none of the items fitted to a theoretical prediction. Thus, for a realistic binomial response model it is necessary to supplement it with one more parameter which corresponds to a certain amount of random noise added to the answers. The second principle is systematic bias which expresses itself in the selective preferences of one response category over all others. It is impossible to predict distribution of responses on these quite numerous items with binomial or any other continuous cumulative function since some response frequencies are out of theoretically permissible limits. Only viable option how to explain the response pattern on these items is to propose that in addition to a general probability *p*_B_ to endorse this statement there is an inclination to choose or avoid one response category which could happen in a certain number of cases. The introduction of an *ad hoc* variable is, no doubt, an inelegant solution. But a very costly alternative is to replace biased items in all popular personality questionnaires with new items that agree better with a theoretical response curve.

Like numerous previous studies of various personality questionnaires (Rost, 1990, 1991; Bolt et al., 2001; Asparouhov and Muthen, 2008; Maij-de Meij et al., 2008; Muthen and Asparouhov, 2009) this study demonstrated that one of the most sophisticated and widely used personality instruments NEO-PI contains a very large number of items which are understood differently by various groups of participants. However, the fact that 87–90% of all items demonstrated improvement when 1PBM was replaced with 3PBM does not automatically speak in favor of two sufficiently large groups of participants who understand items differently. However, there is little reservation that items with two prominent response peaks (see Figure 5) are understood differently by two distinct subgroups of respondents: one group of participants preferred one end of the response scale while others selected answers on the opposite end of the scale. It is less likely that items with a more uniform response pattern (all response alternatives are chosen more uniformly) indicate necessarily for two or more different subsamples of respondents. Two equally weighted binomial distributions with two sufficiently separated probabilities could be just a technical trick with which to approximate a distribution which resists approximation by a single binomial function. Unexpectedly, a substantial number of items had V-type response pattern distribution and it is unrealistic to assume that one of the most elaborated personality measurement instruments available NEO-PI contains 30–40% items which are not understood identically enough by the participants. It is more likely that a characteristic V-shape speaks on numerous occasions about another form of response bias—a pervasive tendency to use a middle category (“neutral” or R3) less frequently than it could be normally expected. Nobody seems to know whether the underuse of the middle response category is a distinctive feature of personality questionnaires or it is an attribute of odd-numbered Likert response scales in general (cf. Kulas et al., 2008; Kulas and Stachowski, 2009).

It is largely acknowledged that application of the parametric IRT models to personality data is still problematic (Reise and Waller, 1993; Reise and Henson, 2003). There is evidence that individuals can respond differently to personality items than to ability test items, making IRT application to personality data even more challenging (Chernyshenko et al., 2001). Almost all popular and widely used personality questionnaires contain items which deviate from the common response pattern and are therefore recommended to be removed (Spence et al., 2012). It is not rare that scales defined as one-dimensional are in fact related to more than one latent trait (Reise and Waller, 2003). The parametric models, particularly normal and logistic, do not approximate personality and psychopathology scales very well sometimes producing misleading results (Meijer and Baneke, 2004). The basic IRT assumption that some personality items represent more extreme expressions of a trait than others (the so-called item “difficulty”), and that this pattern of endorsement needs to be identical across individuals, is a premise that has yet to be proven (Reise and Waller, 2003). Many personality items and even scales may be qualitatively different for different groups of persons suggesting that unidimensional IRT models are not appropriate for their analysis (Egberink et al., 2010). All these evidences may create a misleading impression that the way how personality is typically measured by NEO-PI or other omnibus personality questionnaires is fundamentally flawed. More realistic, however, is a viewpoint that the currently used IRT models need to be revised in order to describe adequately how persons answer personality items including Likert scale questions. One of these needed revisions may be a systematic comparison of alternative response models with the habitual normal or logistic IRT models. Of course, it is unrealistic to expect that researchers will start immediately to use binomial model when it turns out that the binomial model provides more precise approximation to the observed distribution of answers. The advantage of universal and dimensionless probability values *p*_B_ may be relatively small compared with the default options implemented in all available IRT statistical packages. However, those who intend to devise new instruments or revise existing ones may find ideas presented in this paper stimulating. First of all, it could serve as a tool for identifying potential items which are seriously biased. Above I proposed a simple rule how to identify biased items. Shortly repeating the principle, any item can be suspected of being biased if one of its response categories attracts substantially, say 10%, more or less answers than could be theoretically predicted. Another intriguing twist would be to use differences between observed and predicted response frequencies as the weight with which indices of response bias can be computed. For example, the multiplication of answers with social desirability ratings is a convenient way to construct an index of social desirability (Konstabel et al., 2006). Analogously, the disparity between observed and predicted response probabilities could serve as a method for computing different response biases, such as the extreme or middle category response biases.

Applying the binomial latent variable approach allowed us to look at personality measures from a slightly new angle. Although self- and observer-ratings are very similar and agreement between them is substantial (Allik et al., 2010a; Allik et al., 2010b,c), there are still small but cross-culturally replicable differences between these two perspectives (Allik et al., 2010a). The presented data demonstrated that other-ratings are systematically more regular than self-ratings in terms of their binomial fit. This also means that, in the other-report format, there were fewer items which were strongly biased. Does it mean that, in judging someone's personality, judges are less vulnerable to social desirability bias compared to when they have to make similar judgements about themselves? I have no answer to this question. What may be instructive was indirect evidence that other-ratings may be less subject to random answering than self-ratings. Besides determining the test-retest unreliability, this was a novel but indirect way in which to estimate the level of noise in personality ratings. Assuming that this method was comprehensive, the results provided another clue to the riddle why the measurement of some personality traits such as Neuroticism and Conscientiousness are more vulnerable to the influence of noise, that is, random answering, while answers to Openness items are closest to an ideal binomial or mixed-binomial distribution. The strength of any new idea, besides other important qualities, is the number of new problems it could raise. It seems that the mixed-binomial model has fulfilled this promise, since there are a number of new intriguing questions begging for answers.

### Conflict of interest statement

The author declares that the research was conducted in the absence of any commercial or financial relationships that could be construed as a potential conflict of interest.

## Footnotes

^1^One can imagine how confusing it is to explain what the item's “difficulty” is when a typical personality item has a wording “I'm easy-going.”
